# Integrative analysis of the gut microbiota, bile acid pathways, and immune dysregulation in dyslipidemia models

**DOI:** 10.1016/j.isci.2025.114001

**Published:** 2025-11-10

**Authors:** Jiayue Xia, Yinqi Shao, Boxuan Li, Tianyu Wu, Zhi He, Zhiyuan Feng, Zhenzheng Zhang, Shiyu Yin, Yuanyuan Wang, Junhui Yu, Jiongnan Wang, Guiju Sun

**Affiliations:** 1 Key Laboratory of Environmental Medicine and Engineering of Ministry of Education, School of Public Health, Southeast University, Nanjing 210009, China; 2 Department of Nutrition and Food Hygiene, School of Public Health, Southeast University, Nanjing 210009, China; 3 Zhongda Hospital Southeast University, Gulou District, Nanjing 210009, China

**Keywords:** Human metabolism, Immune response, Microbiome

## Abstract

Emerging evidence suggests a link between gut microbiota, bile acid metabolism, and immune dysregulation in dyslipidemia. Through a case-control study involving 140 participants and an animal experiment, we identified significantly elevated counts of peripheral blood CD3^+^ T cells, CD4^+^ T cells, CD8^+^ T cells, and CD19^+^ B cells in patients with dyslipidemia, alongside increased specific bile acids in fecal samples and distinct alterations in gut microbiota composition. The animal model confirmed changes in gut microbiota, bile acid metabolism, and percentage of specific lymphocyte subsets. Also, we identified downregulated hepatic expressions of bile acid metabolism-related proteins in hyperlipidemic rats. Integrated analysis suggested potential associations among gut microbiota, bile acid pathways, and immune dysregulation. Overall, these data highlight the critical role of the potential gut microbiota-bile acid metabolism-immune axis in dyslipidemia, providing potential therapeutic targets for diseases associated with lipid metabolism disorders.

## Highlights

• Patients with dyslipidemia showed altered peripheral blood lymphocyte subsets
• Altered gut microbiota composition correlates with bile acid dysregulation
• Immune dysregulation in dyslipidemia is accompanied by bile acid dysregulation
• Targeted immune modulation may be a therapeutic strategy for lipid metabolism

## Graphical abstract

**Figure:**
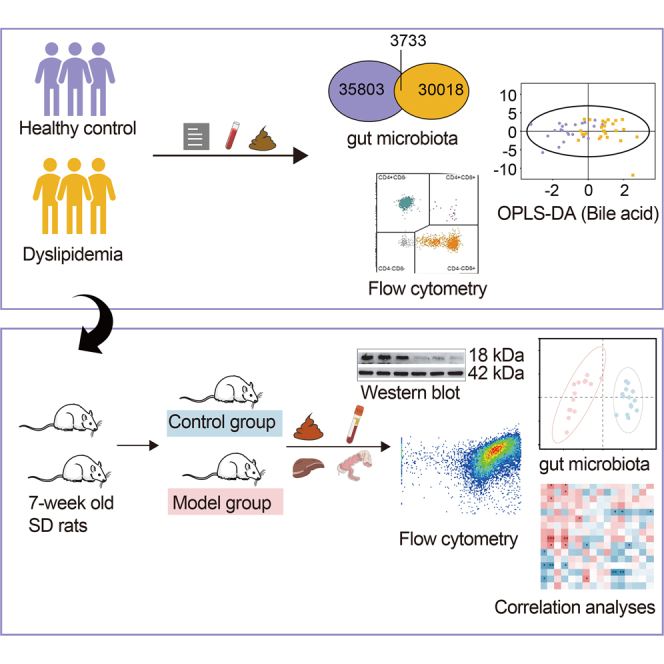

## Introduction

Dyslipidemia represents a significant risk factor for cardiovascular diseases, primarily characterized by abnormal lipid profiles including elevated total cholesterol (TC), low-density lipoprotein cholesterol (LDL-C), and triglycerides (TG), as well as reduced high-density lipoprotein cholesterol (HDL-C) levels.^1^ As global economies experience unprecedented growth, accompanied by profound shifts in dietary patterns and the proliferation of sedentary lifestyles, the worldwide prevalence of dyslipidemia exhibits a concerning and persistent upward trend.^2^ Epidemiological data reveals that the prevalence of dyslipidemia in South Korea experienced a dramatic increase over a fourteen-year period, rising from 7.3% in 2005 to 16.6% by 2019.^3^ According to the 2017 China Cardiovascular Health Index, a substantial proportion approximately, 33.7% of the adult Chinese population was affected by dyslipidemia,^4^ with similar trends observed in Indonesia, Thailand, and Malaysia.^5^ Despite this escalating public health burden, the rates of awareness, treatment adherence, and effective control of dyslipidemia remain alarmingly insufficient across diverse populations and healthcare systems worldwide. In China, the situation is more concerning, with awareness, treatment, and control rates reported at only 18.2%, 11.6%, and 5.4%, respectively.^6^ These data underscore the urgent need to explore the underlying mechanisms of dyslipidemia and to identify effective strategies for prevention and intervention aimed at reducing cardiovascular mortality and the global disease burden.

The gut microbiota maintains intricate bidirectional relationships with host lipid homeostasis, with substantial evidence establishing its pivotal role in metabolic regulation.^7^^,^^8^^,^^9^^,^^10^^,^^11^ A substantial body of evidence has demonstrated that alterations in gut microbiota can disrupt lipid metabolism through multiple mechanisms, including impairing the intestinal barrier, disturbing immune homeostasis, and inducing inflammatory responses.^12^^,^^13^ Meanwhile, bile acids, representing the principal downstream metabolites in cholesterol catabolism, undergo extensive biochemical modification and are regulated by the gut microbiota through complex enzymatic transformations that significantly alter their physicochemical properties and biological activities throughout the enterohepatic circulation.^14^ The gut microbiota influence bile acid metabolism by modulating the conversion of primary to secondary bile acids and reducing the suppression of bile acid synthesis via the farnesoid X receptor (FXR) signaling pathway in the ileum.^15^ Conversely, bile acids, owing to strong antimicrobial properties, can directly regulate the composition and function of gut microbiota.^16^ The bidirectional interaction between gut microbiota and bile acids is crucial in lipid metabolism as well as in maintaining cholesterol and bile acid homeostasis.^17^^,^^18^ Bile acids serve as pivotal immunomodulatory molecules that orchestrate the functional dynamics and proportional balance of diverse immune cell populations, including macrophages, regulatory T cells, and T helper 17 cells, thereby establishing and maintaining immunological homeostasis not only within the intestinal microenvironment but also systemically throughout the organism.^19^ However, the alterations in lymphocyte subsets and the remodeling of bile acid metabolism that occur in dyslipidemia remain incompletely characterized.

Dyslipidemia exerts modulatory effects on immune function through the alteration of monocyte production, differentiation trajectories, and functional activity, establishing a critical interface between metabolic perturbations and immunological competence.^20^^,^^21^ Moreover, dyslipidemia precipitates profound disruptions in immune cell functionality and orchestrates the establishment of chronic inflammatory states, which collectively compromise and attenuate host immunological surveillance and responsiveness to antigenic challenges.^22^^,^^23^^,^^24^ However, the multifaceted regulatory influences of additional contributory factors, including gut microbiota and bile acid metabolism on immunological function, have yet to be systematically integrated into a comprehensive and unified investigational framework, limiting our holistic understanding of these complex interactions in dyslipidemia. Current studies also lack integrative, multidimensional approaches that combine multi-omics analyses. Here, we performed a case-control study to investigate the characteristics of specific peripheral lymphocyte subsets, the composition of gut microbiota, and bile acid metabolism in dyslipidemia. Concurrently, animal experiments were also conducted to provide theoretical support for understanding the pathogenesis of dyslipidemia. The results of this study are expected to shed light on targeted intervention strategies for metabolic diseases from the perspective of the gut microbiota-bile acid metabolism-immune cell axis.

## Results

### Demographic and clinical characteristics of different groups

**Figure 1:**
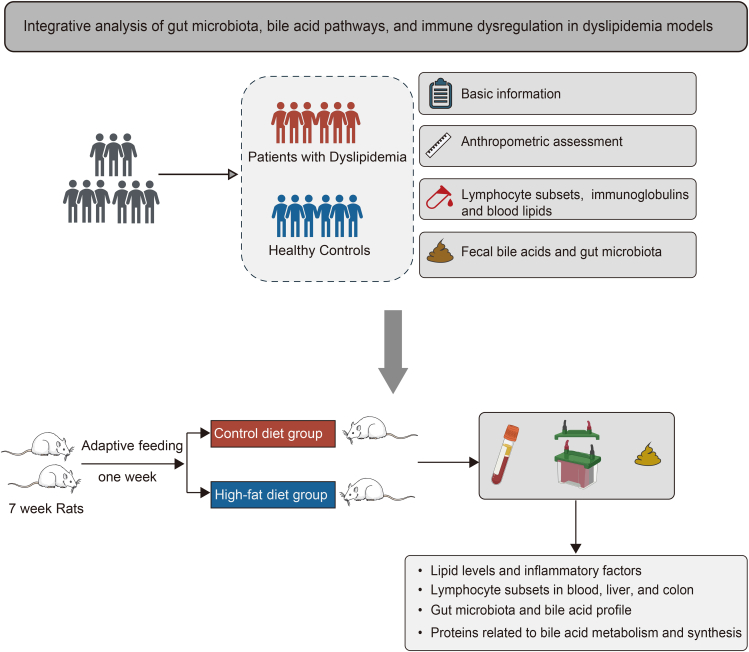
Study design overview

The case-control study included a total of 140 participants, consisting of 70 patients diagnosed with dyslipidemia and 70 healthy controls (Figure 1). In patients with dyslipidemia, serum levels of TC, TG, LDL-C, non-HDL-C, and Apo B were significantly elevated compared to healthy controls, whereas serum Apo A1 levels were significantly reduced (*p* < 0.05) (Table 1). No significant differences were found between groups regarding other demographic characteristics (*p* > 0.05) (Table S1).

**Table 1:** General clinical characteristics of study subjects

	Healthy control (*n* = 70)	Dyslipidemia (*n* = 70)	t/z/χ^2^	*p* value between groups
N (Male/Female)	70 (28/42)	70 (38/32)	2.867	0.090
Age (years)	42.23 ± 9.12	43.46 ± 7.16	−0.887	0.377
BMI (kg/m2)	23.01 ± 2.85	24.69 ± 4.23	−2.765	0.007
Waist-to-Hip Ratio (WHR)	0.87 (0.82, 0.91)	0.88 (0.80, 0.94)	1.098	0.272
Waist-to-Height Ratio (WHtR)	0.50 (0.47, 0.52)	0.51 (0.47, 0.56)	1.196	0.232
Glucose (mmol/L)	4.70 (4.49, 4.98)	4.65 (4.39, 5.03)	−0.404	0.686
Uric Acid (μmol/L)	279.55 (236.00, 328.00)	314.35 (241.00, 406.75)	1.815	0.070
TC (mmol/L)	4.37 ± 0.55	5.58 ± 1.03	−8.648	<0.001
TG (mmol/L)	0.79 (0.61, 1.14)	1.58 (1.00, 2.99)	6.781	<0.001
LDL-C (mmol/L)	2.55 (2.11, 2.91)	3.26 (2.59, 3.65)	5.328	<0.001
HDL-C (mmol/L)	1.52 (1.26, 1.72)	1.25 (0.94, 1.80)	−1.836	0.066
NON-HDL-C (mmol/L)	2.87 ± 0.59	4.13 ± 0.85	−10.205	<0.001
Apo A1 (g/L)	1.45 (1.32, 2.22)	1.37 (1.13, 1.57)	−3.393	<0.001
Apo B (g/L)	0.83 (0.70, 1.04)	1.21 (1.01, 1.73)	6.292	<0.001
Apo A1/Apo B (ratio)	1.75 (1.53, 2.21)	1.08 (0.87, 1.33)	−8.249	<0.001
CRP (mg/L)	0.20 (0.20, 1.39)	0.22 (0.20, 0.82)	−0.186	0.852

BMI, body mass index; TC, total cholesterol; TG, triglycerides; LDL-C, low-density lipoprotein cholesterol; HDL-C, high-density lipoprotein cholesterol; NON-HDL-C, non-high-density lipoprotein cholesterol (this metric is calculated by subtracting high-density lipoprotein cholesterol from total cholesterol); CRP, C-reactive protein. Continuous variables that follow a normal distribution are expressed as mean ± standard deviation, with analysis conducted using an independent two-sample *t* test. Non-normally distributed variables are expressed as the median (P_25_, P_75_), with analysis performed using the Mann-Whitney U test. Categorical variables between the two groups are analyzed using the chi-square test.

### Immunological characteristics of different groups

**Figure 2:**
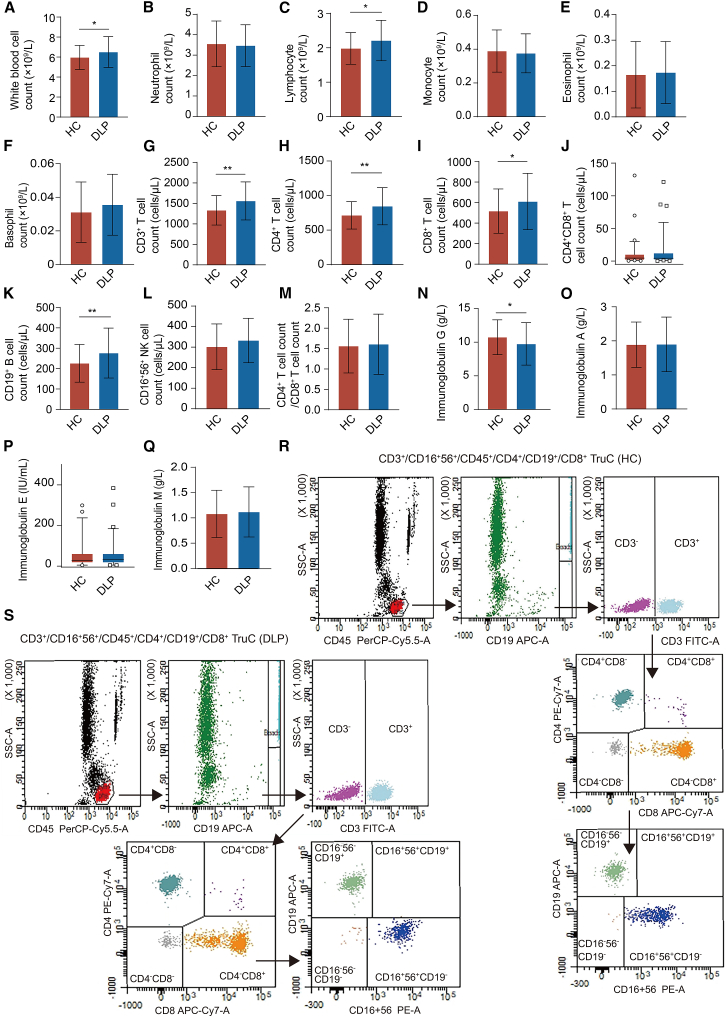
Immunological characteristics of different groups (A) White blood cell count (×10^9^/L). (B) Neutrophil count (×10^9^/L). (C) Lymphocyte count (×10^9^/L). (D) Monocyte count (×10^9^/L). (E) Eosinophil count (×10^9^/L). (F) Basophil count (×10^9^/L). (G) CD3^+^ T cell count (cells/μL). (H) CD4^+^ T cell count (cells/μL). (I) CD8^+^ T cell count (cells/μL). (J) CD4^+^CD8^+^ T cell count (cells/μL). Data are represented as the median (interquartile range). (K) CD19^+^ B cell count (cells/μL). (L) CD16 ^+^ 56^+^ NK cell count (cells/μL). (M) CD4^+^ T cell count/CD8^+^ T cell count. (N) Immunoglobulin G (g/L). (O) Immunoglobulin A (g/L). (P) Immunoglobulin E (IU/mL). Data are represented as the median (interquartile range). (Q) Immunoglobulin M (g/L). (R) Flow cytometry plots represent the proportions of different lymphocyte subsets in the peripheral blood of healthy controls. (S) Flow cytometry plots represent the proportions of different lymphocyte subsets in the peripheral blood of patients with dyslipidemia. All data are represented as the mean ± SD except for CD4^+^CD8^+^ T cell count and Immunoglobulin E. HC: healthy control (*n* = 70), DLP: dyslipidemia (*n* = 70). Differences between the two groups were performed using an independent-samples *t* test for normally distributed data and the Mann-Whitney U test for non-normally distributed data. ∗*p* < 0.05, ∗∗*p* < 0.01, ∗∗∗*p* < 0.001. See also Table S1.

As illustrated in Figures 2A–2F, both the leukocyte count and lymphocyte count were significantly elevated in patients with dyslipidemia compared to healthy controls (*p* < 0.05). There were no significant differences observed between groups concerning the neutrophil count, monocyte count, eosinophil count, and basophil count (*p* > 0.05). Patients with dyslipidemia exhibited significantly elevated counts of CD3^+^ T cells, CD4^+^ T cells, CD8^+^ T cells, and CD19^+^ B cells compared to healthy controls (*p* < 0.05). Nevertheless, the analysis revealed no significant differences between groups in terms of count of CD4^+^CD8^+^ T cells, count of CD16^+^56^+^ NK cells, and count of CD4^+^ T cells/count of CD8^+^ T cells (*p* > 0.05) (Table 2, Figures 2G–2M). The levels of IgG in patients with dyslipidemia were significantly reduced compared to healthy controls (*p* < 0.05) (Figure 2N), while there were no significant differences between groups in levels of IgA, IgM, and IgE (*p* > 0.05) (Figures 2O–2Q). Figures 2R and 2S showed flow cytometry plots representing the proportions of different lymphocyte subsets in the peripheral blood of healthy controls and individuals with dyslipidemia, respectively. The findings indicate altered peripheral blood lymphocyte subset count in individuals with dyslipidemia.

**Table 2:** Comparison of peripheral blood lymphocyte counts between groups

	Healthy control (*n* = 70)	Dyslipidemia (*n* = 70)	t/z	*p* value between groups
CD3^+^ total T cell count	1331.27 ± 358.59	1548.92 ± 507.17	−2.932	0.004
CD4^+^ T cell count	713.79 ± 197.74	827.07 ± 265.09	−2.866	0.005
CD8^+^ T cell count	517.04 ± 215.46	610.61 ± 274.64	−2.243	0.027
CD4^+^CD8^+^ T cell count	4.50 (1.80, 10.07)	4.39 (2.69, 11.79)	0.300	0.764
CD19^+^ B cell count	225.88 ± 92.52	276.34 ± 122.46	−2.751	0.007
CD16^+^56^+^ NK cell count	301.8 ± 111.31	332.59 ± 108.13	−1.656	0.100
CD4^+^ T cell count/CD8^+^ T cell count	1.56 ± 0.66	1.56 ± 0.73	0.011	0.991

For variables that conform to a normal distribution, data are expressed as mean ± standard deviation, and differences between groups are analyzed using the independent samples *t* test. For variables that do not conform to a normal distribution, data are expressed as median (P_25_, P_75_), and differences between groups are analyzed using the Mann-Whitney U test.

### Comparison of bile acid profiles and gut microbiota composition in different groups

**Figure 3:**
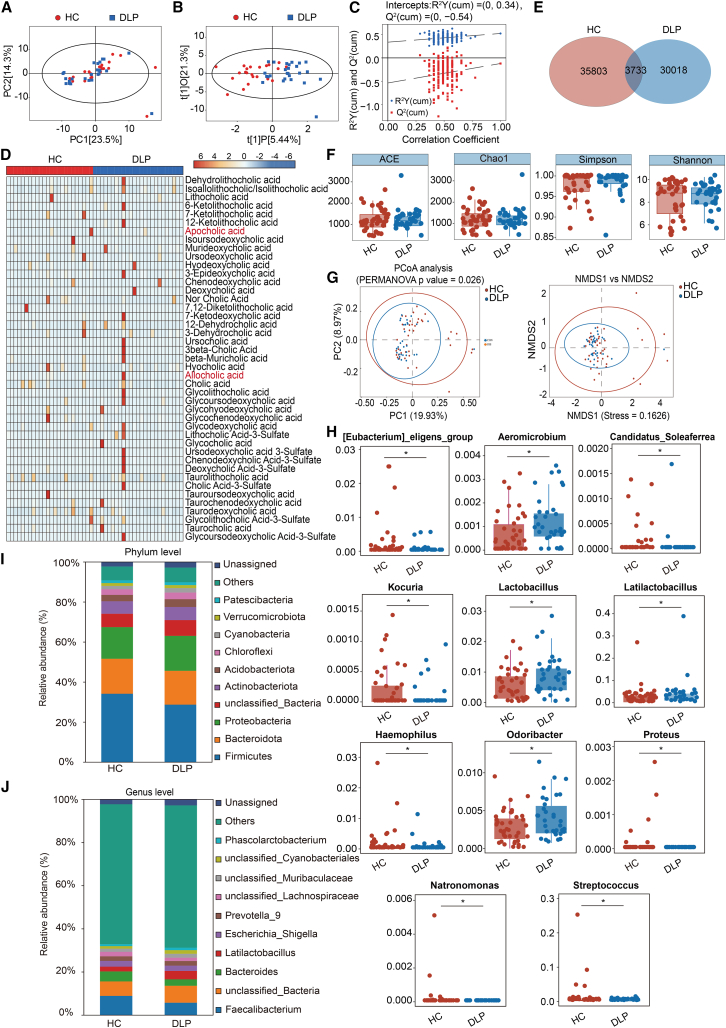
The comparison of bile acid metabolism and gut microbiota of different groups (A) Scatter plot of PCA scores for fecal samples between groups. *n* = 23 from healthy controls and *n* = 26 from patients with dyslipidemia. (B) OPLS-DA analysis score scatterplot of fecal samples between groups. (C) Scatterplot of the results from the permutation test for OPLS-DA model robustness. (D) Heatmap of the levels of different bile acids in all samples from the two groups, with red text indicating significantly different bile acids. The data from the bile acid have been normalized. (E) Venn diagram of ASV characteristics of gut microbiota between groups. (F) Boxplot compares α-diversity between the groups. *n* = 40 from healthy controls and *n* = 33 from patients with dyslipidemia. (G) Beta diversity analysis between groups. Left plot: PCoA based on the unweighted UniFrac algorithm. Right plot: NMDS analysis based on the Unweighted UniFrac algorithm. (H) Relative abundance of differential gut bacterial genera between groups. Data are represented as the median (interquartile range). Differences between the two groups were assessed using the Mann-Whitney U test. (I) Stacked bar plot of species abundance composition at the phylum level. The y axis represents the mean relative abundance (%) of each species within each group. (J) Stacked bar plot of species abundance composition at the genus level. The y axis represents the mean relative abundance (%) of each species within each group. HC: Healthy Control, DLP: Dyslipidemia. ∗*p* < 0.05, ∗∗*p* < 0.01, ∗∗∗*p* < 0.001. See also Table S2.

The analysis of bile acid compounds in fecal samples was conducted utilizing the UHPLC-MS/MS technique. A total of 49 samples were analyzed, comprising 23 from healthy controls and 26 from patients with dyslipidemia. As illustrated in Figure 3A, PCA analysis revealed that the first and second principal component scores accounted for 23.5% and 14.3% of the variance between groups, respectively, with no discernible clustering trend observed in the metabolite profiles. OPLS-DA analysis revealed a significant separation in bile acid profiles, with R^2^X and R^2^Yvalues of 0.268 and 0.522 for the model (Figure 3B). A permutation test over 200 iterations confirmed the robustness of the original model without overfitting (Figure 3C). A total of 43 bile acids were detected in human fecal samples (Figure 3D). Compared to healthy controls, patients with dyslipidemia exhibited significantly elevated levels of apocholic acid and allocholic acid in feces (*p* < 0.05). In addition, there were no significant differences in the levels of free, conjugated, primary, secondary, and total bile acids between healthy controls and patients with dyslipidemia (*p* > 0.05) (Table S2).

Furthermore, we conducted detection and analysis of the gut microbiota in fecal samples. A total of 73 fecal samples were collected from volunteers, comprising 40 samples from healthy controls and 33 samples from patients with dyslipidemia. After high-throughput sequencing, a total of 5,524,024 raw reads were obtained. The final non-chimeric reads, after removing chimeric sequences, amounted to 4,423,137, resulting in an average of about 60,591 reads per sample. OTU clustering analysis produced 85,662 ASVs, corresponding to a total of 4,409,600 reads. Among these, 3,733 ASVs were shared between healthy controls and patients with dyslipidemia (Figure 3E). As shown in Figure 3F, there were no significant differences in alpha diversity indices (ACE, Chao1, Simpson, and Shannon) between groups (*p* > 0.05). However, there are significant differences in the gut microbiota structure between patients with dyslipidemia and healthy controls (*p* < 0.05) (Figure 3G). The first principal coordinate explains 19.93% of the variance, while the second principal coordinate accounts for 8.97%. Further analysis using the unweighted Unifrac algorithm via NMDS confirmed that the structures of the gut microbiota of patients with dyslipidemia and healthy controls are significantly different. No significant differences were observed at the phylum level between groups (*p* > 0.05) (Figure 3I). Compared with healthy controls, the relative abundances of *[Eubacterium]_eligens_group*, *Candidatus_Soleaferrea*, *Kocuria*, *Haemophilus*, *Proteus*, *Natronomonas,* and *Streptococcus* significantly decreased in patients with dyslipidemia (*p* < 0.05). In addition, the relative abundances of *Aeromicrobium*, *Lactobacillus*, *Latilactobacillus,* and *Odoribacter* significantly increased in patients with dyslipidemia when compared with healthy controls (*p* < 0.05) (Figures 3H and 3J). These findings demonstrate that patients with dyslipidemia exhibit significant alterations in gut microbiota composition and bile acid metabolism.

### High-fat diet exposure induces systemic inflammatory responses and an abnormal percentage of lymphocyte subsets in rats

**Figure 4:**
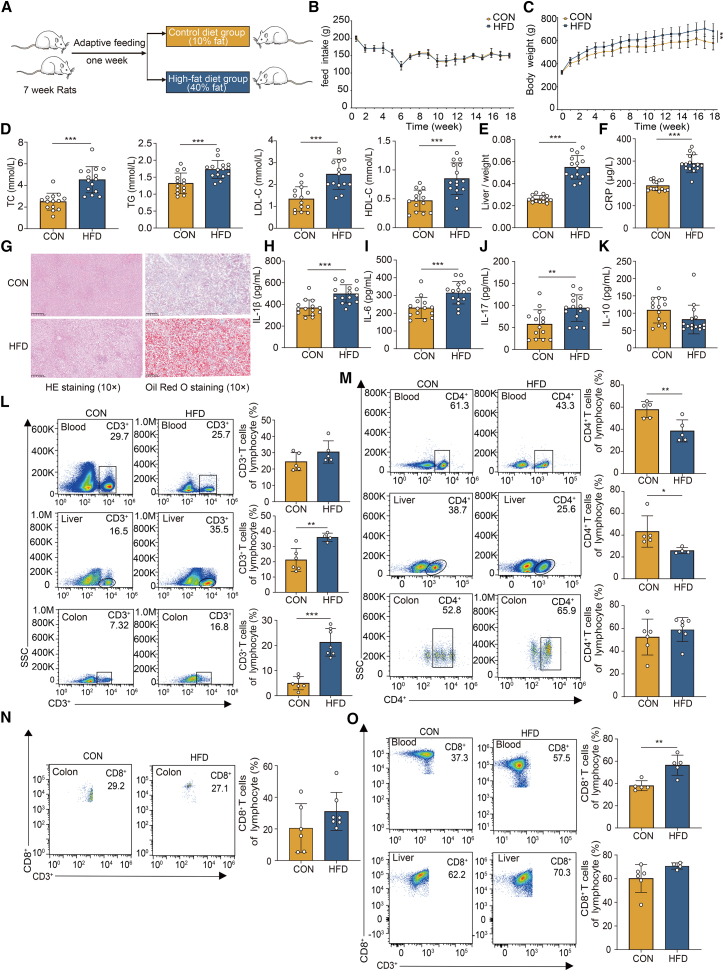
High-fat diet induces systemic inflammatory responses and abnormal percentage of lymphocyte subsets in rats (A) Animal design diagram. *n* = 14 from the normal control group and *n* = 15 from the high-fat model group. (B) Food intake during the modeling period. The data represent the average weekly food intake per rat for each group. (C) Weight change during the modeling period. (D) Serum total cholesterol, triglycerides, low-density lipoprotein cholesterol, and high-density lipoprotein cholesterol levels. (E) The ratio of liver weight to body weight. (F) Serum C-reactive protein. (G) HE staining and Oil red O staining of the rat liver. (H) Serum interleukin-1β. (I) Serum interleukin-6. (J) Serum interleukin-17. (K) Serum interleukin-10. (L) Percentage of CD3^+^ T cells in lymphocytes in the blood, liver, and colon samples. For blood samples, *n* = 5 from the normal control group and *n* = 5 from the high-fat model group; For liver samples, *n* = 6 from the normal control group and *n* = 4 from the high-fat model group; For colon tissue samples, *n* = 6 from the normal control group and *n* = 7 from the high-fat model group. (M) Percentage of CD4^+^ T cells in lymphocytes in the blood, liver, and colon samples. For blood samples, *n* = 5 from the normal control group and *n* = 5 from the high-fat model group; For liver samples, *n* = 5 from the normal control group and *n* = 4 from the high-fat model group; For colon tissue samples, *n* = 6 from the normal control group and *n* = 7 from the high-fat model group. (N) Percentage of CD3^+^CD8^+^ T cells in lymphocytes in colon samples. *n* = 6 from the normal control group and *n* = 7 from the high-fat model group. (O) Percentage of CD3^+^CD8^+^ T cells in lymphocytes in blood and liver samples. For blood samples, *n* = 5 from the normal control group and *n* = 5 from the high-fat model group; For liver samples, *n* = 6 from the normal control group and *n* = 4 from the high-fat model group. CON: Normal control group, HFD: High-fat model group. All data are represented as the mean ± SD. Differences between the two groups were performed using an independent-samples *t* test. ∗*p* < 0.05, ∗∗*p* < 0.01, ∗∗∗*p* < 0.001. See also Table S3.

To further examine the specific alterations in gut microbiota and lymphocyte subsets associated with dyslipidemia, we established an animal model for comprehensive analysis. Following a one-week period of adaptive feeding, 29 rats were randomly assigned to two groups based on body weight. Complete feed formulations were used, with the control group receiving a standard research diet (10% energy from fat) and the model group receiving a high-fat diet (40% energy from fat). Detailed feed ingredient content is shown in Table S3. The design of the animal study is shown in Figure 4A. Changes in food intake and body weight during the modeling period are shown in Figures 4B and 4C. The serum levels of TC, TG, and LDL-C were significantly elevated in the high-fat diet model compared to the low-fat control group (*p* < 0.05). These results indicated the successful establishment of high-fat diet-induced hyperlipidemic rats in the current study (Figure 4D). The liver index in the high-fat model group was significantly elevated compared to the normal control group, with the observed difference reaching statistical significance (*p* < 0.05) (Figure 4E). HE staining revealed that, in comparison to the normal control group, the liver tissue of the high-fat model group exhibited steatosis, characterized by dispersed distribution throughout the liver, hepatocellular swelling, significant adipocyte damage, and cytoplasmic droplet accumulation. Fat vacuoles were readily observable within the cytoplasm, alongside inflammatory alterations in the hepatocytes. Complementary Oil Red O staining further confirmed the presence of lipid accumulation in the liver of rats in the high-fat model group (Figure 4G). Serum levels of CRP, IL-1β, IL-6, and IL-17 were significantly elevated in the high-fat diet model group compared to the normal control group (*p* < 0.05). However, no significant difference was observed in the levels of IL-10 between groups (*p* > 0.05) (Figures 4F, 4H–4K).

Subsequently, flow cytometry was employed to assess the percentage of CD3^+^ T cells of lymphocytes within the blood, liver, and colon tissues of rats. In the high-fat diet model group, the percentage of CD3^+^ T cells of lymphocytes in the blood, liver, and colon tissues appeared to be elevated compared to the normal control group. However, statistically significant differences were observed exclusively in the liver and colon tissues (*p* < 0.05) (Figure 4L). Furthermore, the high-fat model group exhibited a significantly lower percentage of CD4^+^ T cells of lymphocytes in both blood and liver tissues compared to the normal control group (*p* < 0.05). However, no significant difference was observed in the colon tissues (*p* > 0.05) (Figure 4M). The percentage of CD8^+^ T cells in the lymphocytes was significantly elevated in the blood in the high-fat model group compared to the normal control group (*p* < 0.05). Conversely, although the percentage of CD8^+^ T cells of lymphocyte in the liver and colon tissues was higher in the high-fat model group than in the normal control group, these differences did not reach statistical significance (*p* > 0.05) (Figures 4N–4O). The findings suggest that the high-fat diet elicited dysregulation of the percentage of T cells in lymphocyte subsets in rats, which was associated with an enhanced inflammatory response.

### High-fat diet exposure disturbs bile acid metabolism and alters gut microbiota composition in rats

**Figure 5:**
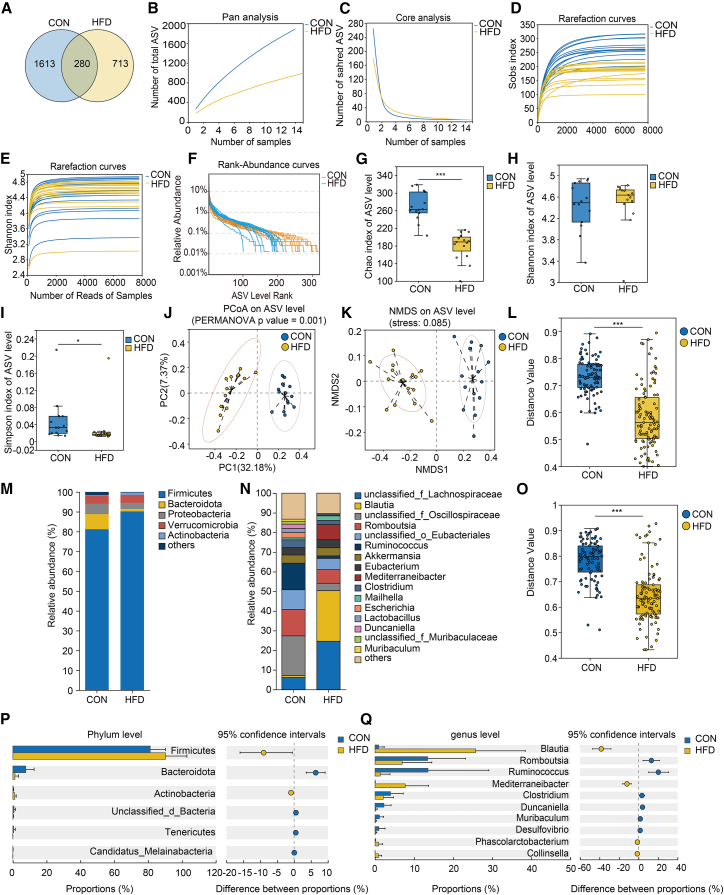
Comparison of the gut microbiota structure in rat models (A) Venn diagram of ASV features in two groups. *n* = 14 from the normal control group and *n* = 15 from the high-fat model group. (B) Pan-species curves. (C) Core species curves. (D) Observed species richness rarefaction curve. (E) Rarefaction curve of the Shannon index. (F) Rank-abundance curve. (G) Chao index. Data are represented as the median (interquartile range). Differences between the two groups were assessed using the Mann-Whitney U test. (H) Shannon index. Data are represented as the median (interquartile range). Differences between the two groups were assessed using the Mann-Whitney U test. (I) Simpson index. Data are represented as the median (interquartile range). Differences between the two groups were assessed using the Mann-Whitney U test. (J) Scatter plot of PCoA based on the unweighted unifrac algorithm. (K) NMDS scatterplot based on the unweighted unifrac algorithm. (L) Beta diversity comparison based on the abund_jaccard algorithm. Data are represented as the median (interquartile range). Differences between the two groups were assessed using the Mann-Whitney U test. (M) Bar chart of gut microbiota species abundance at the phylum level. The y axis represents the mean relative abundance (%) of each species within each group. (N) Bar chart of gut microbiota species abundance at the genus level. The y axis represents the mean relative abundance (%) of each species within each group. (O) Beta diversity comparison based on the Bray-Curtis algorithm. Data are represented as the median (interquartile range). Differences between the two groups were assessed using the Mann-Whitney U test. (P) Differential gut microbiota at the phylum level. The right-hand side of the figure shows the results of the differential analysis between groups, expressed as the mean difference with a 95% confidence interval (95% CI). (Q) Differential gut microbiota at the genus level. The right-hand side of the figure shows the results of the differential analysis between groups, expressed as the mean difference with 95% confidence interval (95% CI). CON: Normal control group, HFD: High fat model group. ∗*p* < 0.05, ∗∗*p* < 0.01, ∗∗∗*p* < 0.001.

Furthermore, the composition of the intestinal microbiota was analyzed in fecal samples from rats. The comprehensive analysis revealed that the total number of optimized sequences across all samples amounted to 1,472,136 pairs, while the cumulative number of optimized bases reached 2,194,533,757 pairs. The mean sequence length across all samples was calculated to be 43,231.36. Following the application of advanced noise reduction techniques, the total number of ASVs identified across all samples was reduced to 6469, with an average of 223.069 per sample. Post-noise reduction, the aggregate number of sequences for all samples was 303,087 pairs. The Venn diagram analysis indicated that 280 ASVs were shared between the normal control group and the high-fat model group (Figure 5A). As the volume of randomly selected sequencing data increased, the observed number of species exhibited a plateau, suggesting that the sequencing data volume was adequate (Figures 5B–5E). Both groups exhibit greater species richness and evenness (Figure 5F). In comparison to the normal control group, the high-fat model group exhibited significantly reduced values for the Chao index, Ace index, and Simpson index (*p* < 0.05). However, no significant difference was observed between groups concerning the Shannon index (*p* > 0.05) (Figures 5G–5I). The intestinal microbiota structures of rats in the high-fat model group and normal control group were significantly different (distance algorithm using binary_bray_curtis, based on PERMANOVA analysis, *p* < 0.05), with the first principal coordinate explaining 32.18% of the degree of variation and the second principal coordinate explaining 7.37% of the degree of variation (Figure 5J). NMDS analysis based on the Unweighted Unifrac algorithm further demonstrated the variability in the structure of the intestinal microbiota of the two groups (Figure 5K). The analysis of beta diversity differences between groups, utilizing the abund_jaccard-based and bray_curtis-based algorithms, revealed statistically significant distinctions between groups (*p* < 0.05) (Figures 5L and 5O).

Figures 5M and 5N demonstrate the colony composition at the phylum and genus level for both groups. At the phylum level, for the normal control group, the top five phyla were *Firmicutes* (81.19%), *Bacteroidota* (7.60%), *Proteobacteria* (5.27%), *Verrucomicrobia* (4.17%), and others (1.53%). For the high-fat model group, the top five phyla were *Firmicutes* (90.20%), *Verrucomicrobia* (4.09%), *Proteobacteria* (3.07%), *Bacteroidota* (1.21%), and *Actinobacteria* (1.14%). At the genus level, the results showed that for the normal control group, the top five genera at the genus level were *unclassified_f_Oscillospiraceae* (20.29%), *Romboutsia* (13.55%), *Ruminococcus* (13.45%), others (13.23%), and *unclassified_o_Eubacteriales* (10.05%). For the high-fat model group, the top five genera at the genus level included *Blautia* (25.74%), *unclassified_f_Lachnospiraceae* (24.60%), others (10.42%), *Mediterraneibacter* (7.72%), *Romboutsia* (6.93%), and others. To further analyze the specific differential colonies between groups, differential analysis was performed at the phylum and genus levels, respectively. Figure 5P shows the differential bacterial phyla at the phylum level, which showed that the *Firmicutes and Actinobacteria* were significantly higher in the high-fat model group compared to the normal control group (*p* < 0.05), while the relative abundance of *Bacteroidota*, *Unclassified_d_Bacteria*, *Tenericutes*, and *Candidatus_Melainabacteria* was significantly lower in the high-fat model group (*p* < 0.05). At the genus level, the genera including *Blautia*, *Mediterraneibacter*, *Phascolarctobacterium*, and *Collinsella* were significantly higher in the high-fat model group compared to the normal control group (*p* < 0.05), while the relative abundance of *Romboutsia*, *Ruminococcus*, *Clostridium*, *Duncaniella*, *Muribaculum*, and *Desulfovibrio* were significantly lower in the high-fat model group compared to the normal control group (*p* < 0.05) (Figure 5Q).

**Figure 6:**
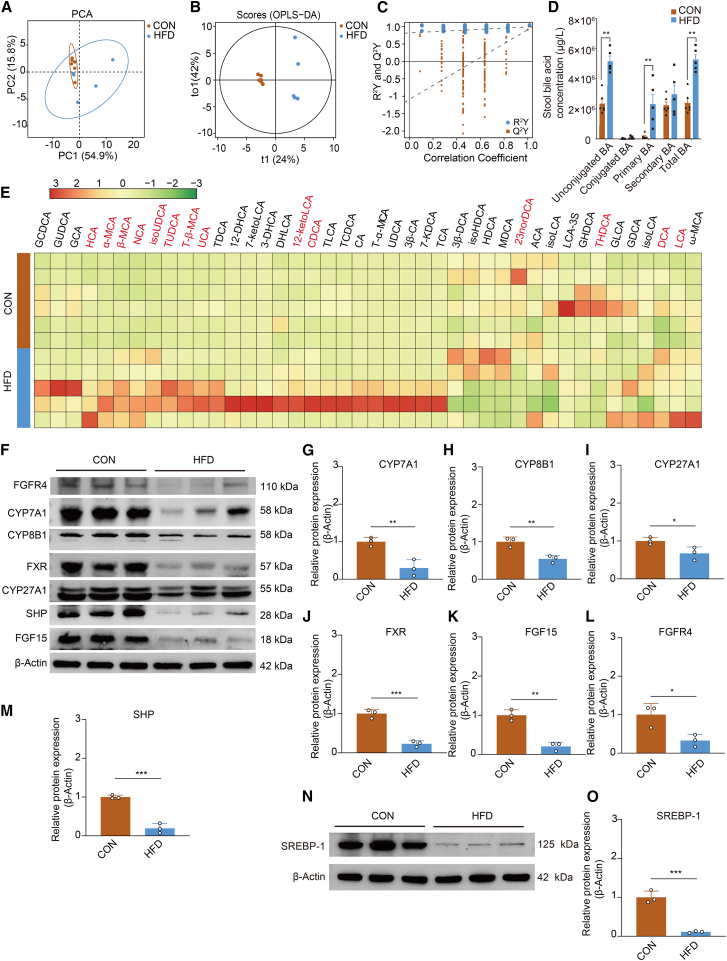
Fecal bile acid and changes in bile acid metabolism-related proteins (A) PCA score plot of fecal samples among groups. *n* = 6 from the normal control group and *n* = 5 from the high-fat model group. (B) OPLS-DA score plot of fecal samples among groups. (C) Permutation test of the OPLS-DA model. (D) Bar graph shows the content of classified bile acids in fecal samples. Data are presented as mean ± SEM. Differences between the two groups were assessed using the Mann-Whitney U test. (E) Heatmap of bile acid content in fecal samples from two groups. Heatmap shows normalized bile acid concentrations in fecal samples. Red indicates bile acids with statistically significant differences between groups. (F) Western blot was used to detect the expression of CYP7A1, CYP8B1, CYP27A1, FXR, FGF15, FGFR4, and SHP in liver tissues. (G) Relative expression of CYP7A1 protein in liver tissues. (H) Relative expression of CYP8B1 protein in liver tissues. (I) Relative expression of CYP27A1 protein in liver tissues. (J) Relative expression of FXR protein in liver tissues. (K) Relative expression of FGF15 protein in liver tissues. (L) Relative expression of FGFR4 protein in liver tissues. (M) Relative expression of SHP protein in liver tissues. (N) Western blot was used to detect the expression of SREBP-1 protein in liver tissues. (O) Relative expression of SREBP-1 protein in liver tissues. CON: Normal control group, HFD: High fat model group. Data for the relative expression of protein are represented as the mean ± SD. Differences between the two groups for the relative expression of protein were performed using an independent-samples *t* test. ∗*p* < 0.05, ∗∗*p* < 0.01, ∗∗∗*p* < 0.001.

Utilizing the UHPLC-MRM-MS/MS platform, a comprehensive analysis was conducted on 11 samples to quantify 50 targeted bile acid metabolites. The sample set comprised 6 cases from the normal control group and 5 cases from the high-fat model group. PCA analysis showed that the first and second principal component scores between groups were 54.91% and 15.78%, respectively, and the clustering trend of metabolite profiles was not significant between groups (Figure 6A). OPLS-DA analysis showed a more significant separation of the clustering trend of bile acid profiles between groups (Figure 6B). As shown in Figure 6C, the model was tested for overfitting through 200 substitutions, and as the retention of substitution gradually decreased, the proportion of Y variables substituted gradually increased, the Q^2^ of the stochastic model gradually decreased, and the intercept between the regression line and the vertical axis of the Q^2^ was less than zero, which indicated that the original model had good robustness of fit and there was no overfitting phenomenon.

In the high-fat model group, the concentrations of free bile acids, primary bile acids, and total bile acids were significantly elevated compared to the normal control group (*p* < 0.05) (Figure 6D). Differential bile acids were screened based on *p* less than 0.05; a total of 14 differential bile acids were screened. Lithocholic acid, 12-ketolithocholic acid, chenodeoxycholic acid, deoxycholic acid, ursocholic acid, α-muricholic acid, β-muricholic acid, hyocholic acid, tauroursodeoxycholic acid, isoursodeoxycholic acid, nor cholic acid, tauro β-muricholic acid were significantly up-regulated in the high-fat model group compared to the normal control group. In addition, two bile acids were significantly down-regulated in the high-fat model group compared to the normal control group, specifically 23-nordeoxycholic acid and taurohyodeoxycholic acid. These findings indicate the significant dysregulation of intestinal microbiota composition and bile acid metabolic pathways in hyperlipidemic rats.

### High-fat diet exposure triggers the dysregulated expression of hepatic bile acid-related proteins in rats

Additionally, we conducted an analysis of the expression levels of proteins associated with bile acid synthesis in the liver. In comparison to the normal control group, the relative expression levels of CYP7A1, CYP8B1, and CYP27A1 proteins were reduced in the hepatic tissues of rats from the high-fat model group (*p* < 0.05) (Figures 6F–6I and S1). We also analyzed the expression of proteins related to the regulation of liver-associated bile acid metabolism. Compared with the normal control group, the relative expression of hepatic FXR protein, FGF15 protein, FGFR4 protein, SHP protein, and SREBP-1 protein was lowered in rats from the high-fat model group (*p* < 0.05) (Figures 6F, 6J–6O, and S1). These findings reveal significant disruptions in the hepatic bile acid metabolism pathway.

### Association between gut microbiota, bile acid metabolism, lymphocyte subsets, and lipid profiles

**Figure 7:**
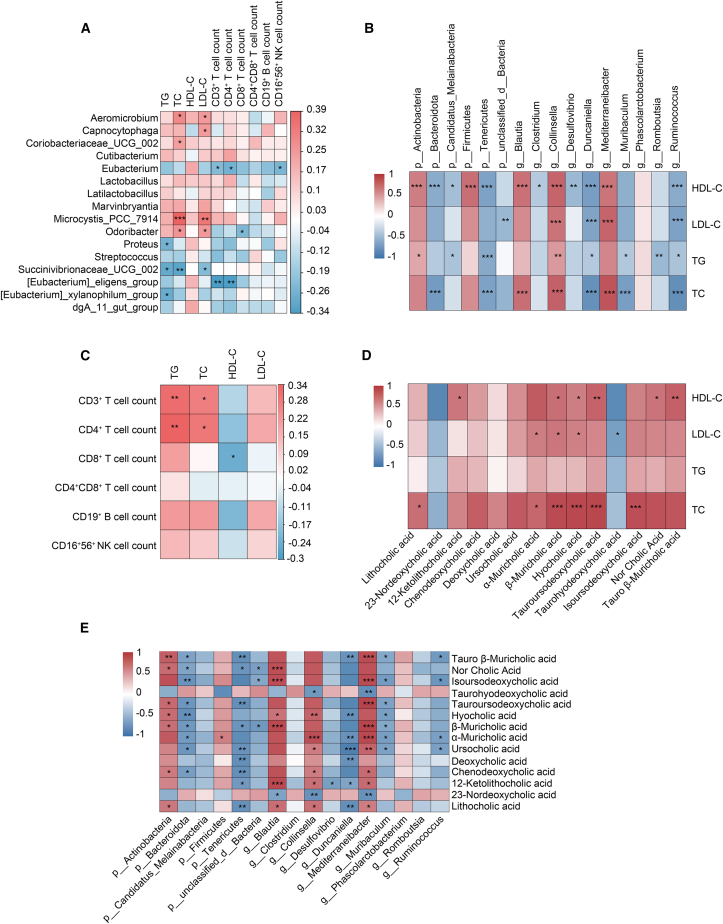
Spearman correlation analysis heatmap of gut microbiota, bile acid, lymphocyte subsets, and blood lipids (A) Heatmap of association between gut microbiota and clinical biochemical parameters in human subjects. (B) Heatmap of association between gut microbiota and blood lipids in rat fecal samples. (C) Heatmap of association between blood lipids and lymphocyte subsets count in human subjects. (D) Heatmap of association between bile acid and blood lipid in rat fecal samples. (E) Heatmap of association between gut microbiota and bile acid profiles in rat fecal samples. Correlation analysis was performed using Spearman's rank correlation. ∗*p* < 0.05, ∗∗*p* < 0.01, ∗∗∗*p* < 0.001.

In human studies, we performed Spearman correlation analyses between fecal gut microbiota, bile acid profiles, blood lipids, and peripheral blood lymphocyte subsets. Our results demonstrated that the genus *Aeromicrobium*, *Microcystis_PCC_7914*, *Odoribacter,* and *Succinivibrionaceae_UCG_002* exhibited significant positive correlations with serum TC and serum LDL-C levels (*p* < 0.05). The genus of *Eubacterium* and *[Eubacterium]_eligens_group* showed significant negative correlations with CD3^+^ T cell count and CD4^+^ T cell count (*p* < 0.05) (Figure 7A). Results from animal studies revealed significant associations between most gut microbiota and blood lipid parameters (*p* < 0.05) (Figure 7B). Additionally, human peripheral blood CD3^+^ T cell count and CD4^+^ T cell count demonstrated significant positive correlations with serum TG and serum TC levels (*p* < 0.05) (Figure 7C). Results from animal studies showed that certain fecal bile acids were significantly positively correlated with blood lipids (*p* < 0.05) (Figure 7D) and the majority of gut microbiota exhibited significant associations with fecal bile acids (*p* < 0.05) (Figure 7E). Similar phenomena were observed in human data (*p* < 0.05) (Figure S2). These findings suggest potential associations among gut microbiota, bile acid metabolism, and lymphocyte subsets in patients with dyslipidemia. These preliminary observations provide a foundation for the further exploration of the multidimensional characteristics of dyslipidemia.

## Discussion

Gut microbiota and its metabolites, especially bile acids, play a crucial role in a variety of physiological processes such as nutrient uptake, metabolic regulation, physiological homeostasis, and host immune defense.^25^ Bile acids are key mediators of metabolism and immune regulation, affecting liver and intestinal function. Bile acid homeostasis is maintained through enterohepatic circulation and is essential for immunometabolic balance.^26^ Our study unveiled intricate bile acid metabolism and immunological dynamics in patients with mild dyslipidemia, highlighting complex interactions between bile acid metabolism and immune system regulation in dyslipidemia. The data presented here offer a perspective on the immunological mechanisms of dyslipidemia, emphasizing the interconnected nature of immune cells, gut microbiota, and bile acid metabolism in patients with dyslipidemia.

Cholesterol metabolism not only affects circulating cholesterol levels but also directly regulates the activity and function of immune cells.^27^ Patients with dyslipidemia demonstrated notable changes in lymphocyte subsets. The significant increase in the count of CD3^+^ T cells, accompanied by elevated counts of CD4^+^ T cells and CD8^+^ T cells, suggests an adaptive immune response intricately linked to lipid metabolic dysregulation. A remarkable finding was the significant reduction in the level of IgG, which may reflect underlying immune dysfunction or compensatory mechanisms. No abnormal changes were observed in the count of CD16^+^56^+^ NK cells in dyslipidemia. These observations open insights into understanding how metabolic perturbations influence antibody-mediated immune responses and potentially contribute to inflammatory processes associated with dyslipidemia. Dyslipidemia-induced low-grade chronic inflammation predominantly activates adaptive immunity.^28^ NK cells, characterized by inherent immune surveillance capacity, maintain a remarkably stable population size under homeostatic conditions, with their numbers tightly regulated in the absence of acute inflammatory stimuli or malignant transformation signals.^29^ Here, we show that the dyslipidemic immune microenvironment is a chronic antigenic stimulus that mainly drives the expansion of the count of T cells and B cells, whereas the count of CD16^+^56^+^ NK cells remains unchanged.

Dysbiosis of the gut microbiota represents a critical determinant in the pathogenesis of dyslipidemia, contributing to metabolic perturbations through complex microbiome-host interactions.^30^^,^^31^^,^^32^ Compositional and functional perturbations of gut microbiota critically modulate bile acid metabolism and composition, thereby being instrumental in driving the initiation and progression of metabolic dysregulation.^33^^,^^34^ Environmental perturbations disrupting gut microbiota homeostasis or microbiota-host interactions can substantially impair bile acid biosynthesis and metabolic processing. Notably, the present study found an interesting phenomenon that the relative abundances of *Lactobacillus*, *Lactilactobacillus,* and *Odoribacter* were significantly increased in patients with dyslipidemia. An increase in a specific genus of bacteria may be a compensatory response of the organism to metabolic disorders. In the context of metabolic disorders, certain *lactobacilli* may be involved in lipid metabolism regulation through specific pathways. This observation unveils the intricate microbiome dynamics underlying dyslipidemia, potentially representing a sophisticated host adaptive response to metabolic perturbations, wherein the precise molecular mechanisms remain to be comprehensively elucidated.

Bile acid profiling demonstrated substantially increased concentrations of cholic acid and deoxycholic acid among patients with dyslipidemia. We also observed significant alterations in bile acid metabolism among high-fat dietary intervention models, manifesting as substantial elevations in free bile acids, primary bile acids, and total bile acid concentrations. Intriguing discrepancies in fecal bile acid profiles between human populations and rat models unveil the intricate metabolic adaptation mechanisms, highlighting the complexity of interspecific and individual metabolic responses to nutritional interventions. Cholic acid and deoxycholic acid represent secondary bile acids, generated through the gut microbiota-mediated biotransformation of primary bile acids via enzymatic hydrolysis and dehydroxylation processes, predominantly catalyzed by specific gut microbiota such as Clostridium.^35^ Dyslipidemia is frequently associated with insulin resistance or high-fat dietary patterns, characterized by hepatic nuclear receptor signaling dysregulation, specifically involving the suppression of the FXR-small heterodimer partner (SHP) pathway and subsequent up-regulation of CYP7A1 expression, ultimately precipitating enhanced primary cholic acid biosynthesis.^36^ In a high-fat model, specific bacterial populations exhibiting elevated bile salt hydrolase (BSH) activity preferentially proliferate, thereby expediting the enzymatic transformation of cholic acid to deoxycholic acid. There is a bidirectional regulatory relationship between gut flora and bile acids. On the one hand, the gut microbiota regulates bile acid metabolism and influences the size and composition of the bile acid pool.^37^ On the other hand, bile acids selectively promote or inhibit the growth of specific microbial populations through unique chemical properties and physiological functions, thereby reshaping the composition of the intestinal microbiota.^38^ Advances in genomics and metabolomics have revealed that the pathophysiology of metabolic diseases is regulated by bile acids and their receptors. This finding provides strong evidence for bile acid-based therapeutic strategies and offers perspectives and molecular targets for the diagnosis and treatment of metabolic diseases. Current bile acid-based therapies include agonists/antagonists directly targeting bile acid receptors and indirect modulation of gut flora through gut microbiota-bile acid interactions.^25^

High-fat dietary intervention induces profound microbiome restructuring in animal models. At the phylum level, a significant proliferation of *Firmicutes* and *Actinobacteria* emerges concurrently with substantial reductions in *Bacteroidota*, *Tenericutes*, and *Candidatus_Melainabacteria*, underscoring complex ecological regulatory dynamics within the intestinal microbiota. Genus-level analyses unveil nuanced microbiota population shifts, demonstrating the selective proliferation of specific bacteria. The relative abundance of *Blautia*, *Mediterraneibacter*, *Phascolarctobacterium*, and *Collinsella* exhibits remarkable increases, while the relative abundance of *Romboutsia*, *Ruminococcus*, *Clostridium*, *Duncaniella*, *Muribaculum*, and *Desulfovibrio* demonstrates significant diminution, highlighting the sophisticated ecological niche remodeling induced by high-fat nutritional environments. Notably, the intestinal flora of patients with dyslipidemia showed flora changes inconsistent with animal models. The *Firmicutes* consist of a type of gram-positive bacteria that are typically associated with energy metabolism and fat absorption. The increase of *Firmicutes* may reflect the remodeling of the gut microbiota due to a high-fat diet, allowing these bacteria to have a competitive advantage in a high-fat environment.^39^ The *Bacteroidota* consists of Gram-negative bacteria that primarily participate in the degradation of polysaccharides and complex carbohydrates. It may indicate that a high-fat diet reduces the gut microbiota’s ability to metabolize complex carbohydrates, thereby impacting the host’s energy balance and metabolic health.^40^ The genus *Blautia* plays an important metabolic role in the gut, and its increase may be associated with energy metabolism and fat absorption following a high-fat diet. The decrease in *Romboutsia* may be related to a weakened gut barrier function, which in turn could affect the host’s immune response and overall health.^40^ A high-fat diet significantly altered the structure and function of the gut microbiota in rats, and these changes may be closely related to the host’s metabolic health. The increase in *Firmicutes* and *Actinobacteria*, along with the decrease in *Bacteroidota* and other beneficial phyla, may reflect the remodeling of the gut microbiota due to a high-fat diet, which in turn affects the host’s energy metabolism, inflammatory response, and gut barrier function. Findings from animal experiments provided mechanistic hypotheses, but translation to humans still requires further functional homology validation. Further *in vitro* co-culture and fecal transplantation experiments are needed in the future to explore the core functions of key genera in animals at the human level.

In the current study, the percentage of CD3^+^ T cells, CD4^+^ T cells, and CD8^+^ T cells in blood, liver, and colon tissues changed following high-fat dietary intervention. Bile acids are not only cholesterol metabolites but also metabolic sentinels of the host immune system, which regulate innate and adaptive immune cells. We observed significant increases in serum levels of CRP, IL-1β, IL-6, and IL-17, and significant reductions in the hepatic relative expression of bile acid metabolism-related proteins, including CYP7A1, CYP8B1, CYP27A1, FXR, FGF15, FGFR4, and SHP in high-fat diet models. The high-fat dietary intervention led to the reduced expression of FXR with SHP, which implies a loss of the direct inhibition of NLRP3 inflammatory vesicles by bile acids and sustained release of IL-1β, IL-18, and TNF-α from hepatic macrophages and circulating monocytes.^41^ FXR is activated in macrophages and dendritic cells (DCs), blocking the NF-κB/NLRP3 pathway and reducing pro-inflammatory factors such as IL-1β and TNF-α.^42^ Reduced expression of FGF15 failed to induce the stabilization of SHP phosphorylation via the FGFR4/β-Klotho complex, loss of secondary inhibition of CYP7A1, and continued expansion of the bile acid pool. Subsequently, excess deoxycholic acid/lithocholic acid directly binds to RORγt, further amplifying Th17 and inhibiting Foxp3^+^ Treg.^19^^,^^41^ Intestinal microbiota convert primary bile acids into secondary bile acids through bile salt hydrolase (BSH) and 7α-dehydroxylation reaction, and secondary bile acids then feedback to regulate host immune cells, forming a closed loop of gut microbiota-metabolism-immunity.^19^ Bile acids precisely regulate the function and ratio of immune cells, thus maintaining the immune homeostasis of the intestinal tract and the whole body. The current study only focuses on CD3^+^ T cells, CD4^+^ T cells, and CD8^+^ T cells, and further studies are still needed to explore the role of more types of T and B immune cells in dyslipidemia and the complex relationship between bile acid metabolism and immune cells.

The relative abundance of certain bacteria, including *Aeromicrobium*, *Microcystis_PCC_7914*, *Odoribacter*, and *Succinivibrionaceae_UCG_002,* was significantly and positively correlated with the levels of TC and LDL-C. In addition, there were significant positive correlations between CD3^+^ T cell count, CD4^+^ T cell count, and serum TG and TC. Animal experiments further validated these findings, providing important evidence for interactions between the gut microbiota, bile acid metabolism, and the immune system. Bile acid, gut microbiota, and immune cells are a three-way conversation system. Gut microbiota modifies hepatic primary bile acids into secondary bile acids, thus changing the total amount and type of bile acids. Conversely, bile acids have an antibacterial or pro-bacterial effect, directly shaping the composition and spatial distribution of the flora.^43^ Bile acids and immune cells also influence and regulate each other.^43^ Gut microbiota determines the chemical language of bile acids, while bile acids act as signal messengers to translate the state of gut microbiota into instructions for immune cells, which then regulate the ecology of gut microbiota. The three constitute a continuous cycle of gut microbiota-bile acid metabolism-immune cell, and any imbalance will affect the whole system, which in turn affects inflammation, metabolism, tumor immunity, and other pathophysiological processes. The research provides critical insights into the immunological mechanisms of dyslipidemia, emphasizing the interconnected nature of the gut microbiota-bile acid metabolism-immune cell axis. By elucidating these complex interactions, the study offers promising directions for personalized immune and metabolic interventions.

### Limitations of the study

To the best of our knowledge, the present study is a study aimed at exploring gut microbiota, bile acid metabolism, and specific lymphocyte subsets in Chinese dyslipidemia. This study used a multi-dimensional approach and cross-species validation to strengthen the relevance of the findings, offering potential insights into the gut microbiota-bile acid metabolism-immune cells axis as a regulator of lipid homeostasis. Nevertheless, these data are not without limitations. A major limitation of this study is the limited lymphocyte subsets, along with the lack of relevant data on tissue lymphocytes in humans. In current studies, only lymphocyte subsets including CD4^+^ T cells, CD8^+^ T cells, CD4^+^CD8^+^ T cells, CD19^+^ B cells, and CD16^+^56^+^ natural killer cells have been detected in human peripheral blood. Given the functional diversity of immune cell differentiation and the complexity of cell types, a more comprehensive immune cell landscape is still needed to elucidate the characteristic changes in immune cells associated with dyslipidemia. Furthermore, current animal studies have focused primarily on CD3^+^ T cells, CD4^+^ T cells, and CD8^+^ T cells, without exploring the alterations in additional lymphocyte subsets. Furthermore, the heterogeneity in dietary patterns and cultural determinants across diverse geographical populations potentially engenders differential modulations in gut microbiota composition and metabolite profiles. Consequently, large-scale, multi-national cohort studies encompassing varied demographic and cultural contexts are imperative to validate the generalizability and specificity of the observed phenomena.

Another limitation of this study is that the cross-sectional nature of the human study resulted in the inability to infer causality. Additionally, there is still some variability in the metabolism between the rat model of hyperlipidemia dependent on a high-fat diet and human dyslipidemia. Additionally, while current dyslipidemia employs human studies and animal models to explore microbiota profiles, bile acid metabolism, and lymphocyte subsets associated with dyslipidemia, it does not adequately address the spatial-contextual relationship between different immune cell dynamics across diverse anatomical niches and alterations in gut microbiota composition and bile acid metabolic pathways. This limitation is particularly significant considering the substantial heterogeneity and tissue-specific functionality of immune cell populations across different physiological compartments, thereby constraining the comprehensive understanding of the gut microbiota-bile acid metabolism-immune cells axis. Finally, a potential limitation of this study is that it is an exclusive reliance on rat models without considering the deployment of diverse biological systems to explore potential translational implications. Consequently, cross-species validation across multiple model organisms remains imperative to establish the robustness and generalizability of these findings. These insights may ultimately guide the development of therapeutic targets for dyslipidemia and associated chronic diseases within the context of the gut microbiota-bile acid metabolism-immune cells axis.

## Resource availability

### Lead contact

Further information and the datasets generated and analyzed for this study may be directed at and will be fulfilled by the lead contact, Guiju Sun (gjsun@seu.edu.cn).

### Materials availability

This study did not generate new unique reagents.

### Data and code availability

• The raw amplicon sequencing data from rat fecal samples and human fecal samples have been deposited at the National Center for Biotechnology Information (NCBI) and are publicly available as of the date of publication. Accession numbers are listed in the key resources table.
• Bile acid metabolomics data for rat fecal samples and human fecal samples have been deposited at MetaboLights and are publicly available as of the date of publication. Accession numbers are listed in the key resources table.
• This article does not report any original code.
• Any additional information required to reanalyze the data reported in this article is available from the lead contact upon request.

## Acknowledgments

We sincerely thank all the participants for their invaluable time to this study. We are also deeply grateful to the clinical staff at Zhongda Hospital, Southeast University. This research is funded by the National Nutrition Research Fund Program initiated by the 10.13039/100015862Chinese Nutrition Society (CNS-NNSRG2021-39), the 10.13039/501100008081SEU Innovation Capability Enhancement Plan for Doctoral Students (CXJH_SEU 25221 and CXJH_SEU 24212), and the Postgraduate Research & Practice Innovation Program of Jiangsu Province (Grant numbers: KYCX24-0486).

## Author contributions

Conceptualization, J.Y.X., T.Y.W., Z.H., and Z.Y.F.; methodology, B.X.L., Z.Z.Z., Z.H., and T.Y.W.; investigation, J.Y.X., B.X.L., Z.Y.F., Z.Z.Z., and Y.Y.W.; writing – original draft, J.Y.X., Y.Q.S., and B.X.L.; writing – review and editing, J.N.W. and G.J.S.; funding acquisition, G.J.S.; resources, G.J.S.; supervision, Y.Y.W., J.H.Y, and S.Y.Y.

## Declaration of interests

The authors declare no competing interests.

## Declaration of generative AI and AI-assisted technologies in the writing process

During the preparation of this work, the author(s) used Grammarly to edit/improve the English. After using this tool/service, the author(s) reviewed and edited the content as needed and take(s) full responsibility for the content of the publication.

## STAR★Methods

### Key resources table

**Table:** 

REAGENT or RESOURCE	SOURCE	IDENTIFIER
**Antibodies**		

Pacific Blue™ anti-rat CD45 Antibody	Biolegend	Cat#202225; RRID: AB_2721618
Alexa Fluor® 488 anti-rat CD3 Antibody	Biolegend	Cat#201406; RRID: AB_893303
PerCP/Cyanine5.5 anti-rat CD4 Antibody	Biolegend	Cat#201519; RRID: AB_2814097
PE/Cyanine7 anti-rat CD8a Antibody	Biolegend	Cat#201716; RRID: AB_2814100
CYP7A1 Rabbit pAb	ABclonal	Cat#A22897; RRID: AB_3105781
CYP8B1 Rabbit pAb	ABclonal	Cat#A25847; RRID:N/A
CYP27A1 Rabbit mAb	ABclonal	Cat#A23250; RRID: AB_3674587
FGFR4 Rabbit pAb	ABclonal	Cat#A7555; RRID: AB_2768080
FXR/NR1H4 Rabbit mAb	ABclonal	Cat#A24015; RRID:N/A
SHP/NR0B2 Rabbit pAb	ABclonal	Cat#A16454; RRID: AB_2770666
FGF19 Rabbit pAb	ABclonal	Cat#A6589; RRID: AB_2767182
SREBP1 Rabbit Polyclonal Antibody	HUABIO	Cat# HA500210; RRID: AB_3071307
anti-beta Actin Rabbit pAb	Servicebio	Cat#GB11001-100; RRID:N/A

**Biological samples**		

Blood samples from individuals with dyslipidemia and healthy individuals	This paper	N/A
Fecal samples from individuals with dyslipidemia and healthy individuals	This paper	N/A
Fecal samples from rats	This paper	N/A
Blood samples from rats	This paper	N/A

**Critical commercial assays**		

BD Multitest™ 6-color TBNK	BD Biosciences	Cat#662967
Zombie Yellow™ Fixable Viability Kit	Biolegend	Cat#423103
True-Nuclear™ Transcription Factor Buffer Set	Biolegend	Cat#424401
Rat TC ELISA Kit	Nanjing Jiancheng Bioengineering Institute, Nanjing, China	Cat#A111-1-1
Rat TG Assay Kit	Nanjing Jiancheng Bioengineering Institute, Nanjing, China	Cat#A110-1-1
Rat LDL-C Assay Kit	Nanjing Jiancheng Bioengineering Institute, Nanjing, China	Cat#A113-1-1
Rat HDL-C Assay Kit	Nanjing Jiancheng Bioengineering Institute, Nanjing, China	Cat#A112-2-1
Rat C-Reactive Protein (CRP) ELISA Kit	Nanjing Lapuda Biotechnology Co.,Ltd. (Nanjing, china)	Cat#CRP-R001
Rat Interleukin-1β (IL-1β) ELISA Kit	Nanjing Lapuda Biotechnology Co.,Ltd. (Nanjing, china)	Cat#IL1B-R002
Rat Interleukin-6 (IL-6) ELISA Kit	Nanjing Lapuda Biotechnology Co.,Ltd. (Nanjing, china)	Cat#IL6-R003
Rat Interleukin-10 (IL-10) ELISA Kit	Nanjing Lapuda Biotechnology Co.,Ltd. (Nanjing, china)	Cat#IL10-R004
Rat Interleukin-17 (IL-17) ELISA Kit	Nanjing Lapuda Biotechnology Co.,Ltd. (Nanjing, china)	Cat#IL17-R005

**Deposited data**		

Bile acid metabolomics data of rat fecal samples	MetaboLights	MetaboLights: MTBLS13092
Bile acid metabolomics data of human fecal samples	MetaboLights	MetaboLights: MTBLS13093
Raw amplicon sequencing data from rat fecal samples	This paper	NCBI: PRJNA1330056
Raw amplicon sequencing data from human fecal samples	This paper	NCBI: PRJNA1330509

**Experimental models: organisms/strains**		

Sprague-Dawley (SD) rats	Jinan Pengyue Experimental Animal Breeding Co., Ltd., China	N/A

**Software and algorithms**		

R	R Core Team https://www.r-project.org/	R Version 4.4.2
Rstudio	RStudio Team	https://github.com/rstudio/rstudio
Prism 8.0.2 (GraphPad)	https://www.graphpad.com/	N/A
ImageJ	https://imagej.net/ij/download.html	N/A

### Experimental model and study participant details

#### Study design and participants

The overall study design is shown in Figure 1. This case-control study was registered with the Chinese Clinical Trial Registry (Registration number: ChiCTR2500096000, https://www.chictr.org.cn/) and received ethical approval from the Ethics Committee of Zhongda Hospital, Southeast University (approval no. 2024ZDSYLL478-P01). One hundred and forty volunteers (66 males and 74 females) aged 18 to 65 years provided written informed consent and participated voluntarily in this study. Dyslipidemia was defined according to the 2016 Chinese Adult Dyslipidemia Prevention Guideline as: serum TC ≥ 5.2 mmol/L; serum TG ≥ 1.7 mmol/L; serum HDL-C < 1.0 mmol/L or serum LDL-C ≥ 3.4 mmol/L.^44^ The exclusion criteria were: (a) participants with liver, kidney, brain or cardiovascular diseases, or diabetes; (b) pregnant or lactating women, or individuals following special diets (e.g., ketogenic, vegetarian, or calorie-restricted regimens). The case group comprised patients newly diagnosed with dyslipidemia within the previous month who had not received lipid-lowering medications. The control group consisted of healthy individuals from the same organization, with a body mass index (BMI) between 18.5 and 23.9 kg/m^2^ and lipid profiles within normal reference ranges. The case group and the control group had 70 participants in each group.

#### Questionnaires and physical assessment

Baseline participant data were collected through in-person interviews. Personal health information encompassed individual and family medical histories of major chronic conditions, including hypertension, diabetes, dyslipidemia, peptic ulcer, chronic enteritis, chronic nephritis, hematologic disorders, malignancies, and cerebrovascular events. Tobacco consumption status among study participants was stratified into three distinct categories: individuals with no history of tobacco use (non-smokers), those actively engaged in tobacco consumption at the time of assessment (current smokers), and individuals who had previously engaged in tobacco use but had subsequently discontinued the practice (former smokers). Alcohol consumption status was categorized as current drinker, former drinker, or non-drinker. Physical activity was evaluated using the International Physical Activity Questionnaire (IPAQ) that assessed occupational, transportation, exercise, leisure-time, and household activity domains. Dietary intake was assessed using a validated food frequency questionnaire (FFQ), which referenced the B5 Food Coding Manual and included food images to evaluate the frequency and quantity of food group consumption over the past year. Additionally, participants completed three consecutive 24-hour dietary recalls (including two weekdays and one weekend day), and data on dietary supplement use were documented.

Anthropometric and hemodynamic measurements were obtained using standardized protocols, including body weight, height, waist and hip circumferences, and blood pressure. Height was measured using a stadiometer with participants standing barefoot on a flat surface, maintaining an upright posture with standardized positioning: head in the plane, heels, sacrum, and scapulae in contact with the vertical measuring column. Height was recorded to the nearest centimeter when the stadiometer’s headboard was compressed against the participant’s vertex, with measurements reported in meters (m). Body weight was measured simultaneously and recorded in kilograms (kg). Waist and hip circumferences were measured using a flexible tape measure accurate to 0.1 cm, with two readings taken for each and the average used. Waist circumference was measured at the midpoint between the lower margin of the last palpable rib and the top of the iliac crest, while hip circumference was measured at the widest part of the buttocks. Body mass index (BMI) was calculated using the formula: BMI = weight (kg)/height (m).^2^ Blood pressure was measured after participants had rested for at least 10 min in a calm state. Measurements were taken in a seated or supine position with the arm exposed, relaxed, and supported at heart level. Participants were asked to remain still, avoid speaking, and refrain from eating during the measurement. The cuff was positioned at heart level, and systolic and diastolic pressures were recorded. Blood pressure was measured in triplicate with 30 s intervals between measurements, and the arithmetic mean of the three readings was calculated for analysis.

#### Design of animal experiment

Twenty-nine seven-week-old male Sprague-Dawley (SD) rats were selected as experimental animals. The experimental animals were purchased from Jinan Pengyue Experimental Animal Breeding Co. (Laboratory Animal Production License: SCXK (Lu) 2022-0006). Rats were housed in the Specific Pathogen Free-grade animal room. The rearing temperature was 22±2°C, relative humidity was 40%-70%, and the light/dark cycle was 12 hours. Following a one-week acclimatization period, the rats were randomly allocated into two groups based on body weight: a normal control group (n=14) and a model group (n=15). Throughout the 18-week experimental period, all rats were maintained on standardized complete formulated diets. The control group received a standard diet (with 10% of energy derived from fat), while the hyperlipidemia model group was fed a high-fat diet (with 40% of energy derived from fat). The animal experimental protocol was approved by the Animal Ethics Committee of Southeast University (Approval No. 20231017003).

### Method details

#### Blood sample collection and biochemical testing

Human Blood samples were collected and were centrifuged at 3500 rpm for 15 min using a low-speed centrifuge at room temperature. Serum samples from humans were used for the determination of TC, TG, LDL-C, HDL-C, Apo A1, and Apo B, as well as CRP. Residual serum samples were stored at -80°C.

#### Flow-cytometric identification of human peripheral blood lymphocyte subsets

The analysis of peripheral blood lymphocyte subsets was performed by the Clinical Laboratory Department of Zhongda Hospital, Southeast University. The BD FACSCanto™ II flow cytometer (Becton, Dickinson and Company, BD Biosciences, USA) was employed for determining human peripheral blood lymphocyte subsets. Human peripheral blood lymphocyte subpopulation assay combinations were CD3^+^/CD16^+^56^+^/CD45^+^/CD4^+^/CD19^+^/CD8^+^ TruCount Absolute Count Tubes. The specific assessed lymphocyte subsets included count of CD4^+^ T cells, CD8^+^ T cells, CD4^+^CD8^+^ T cells, CD19^+^ B cells, CD16^+^56^+^ natural killer (NK) cells.

#### Detection of immunoglobulins in human blood specimens

The BNII SYSTEM specific protein analyzer (Siemens Healthcare Diagnostics Products GmbH, Germany) was used to measure serum immunoglobulin levels. The tested immunoglobulins included immunoglobulin E (IgE), immunoglobulin A (IgA), immunoglobulin G (IgG), immunoglobulin M (IgM), and complement component C3.

#### Collection and flow-cytometric identification of animal samples

All rats were sampled after 18 weeks of continuous intervention. After isoflurane anesthesia, the rats were dissected, and whole blood was collected using EDTA anticoagulant tubes and standard 15 mL EP tubes. The EP tubes were centrifuged at 3500 rpm for 15 min at 4°C, and the supernatants were subsequently stored at -80°C for subsequent analyses. Serum samples from rats were used for biochemical testing of TC, TG, LDL-C, and HDL-C, CRP, IL-1β, IL-6, IL-10 and IL-17. The serum samples also were used for untargeted metabolomics analysis. Flow cytometry was employed to identify percentage of CD3^+^ T cells, CD4^+^ T cells and CD8^+^ T cells of lymphocyte in the blood, liver and colon tissues. For rat blood samples, a suitable volume of blood sample is diluted, incubated with the corresponding antibody, and subjected to red blood cell lysis. Following washing and resuspension, the sample is analyzed using flow cytometry. For rat liver and colon tissue samples, a single-cell suspension was first prepared. The samples were washed with phosphate-buffered saline (PBS) and then enzymatically digested using digestive enzymes. The enzymatically digested tissue was filtered through a cell filter, and the resulting single-cell suspension was washed with PBS. Finally, the washed single-cell suspension was centrifuged to remove the supernatant, and the cells were resuspended in appropriate culture medium. Cell density was measured using a cell counter, and necessary live-cell staining was performed to assess cell viability. After determining the viability of the corresponding tissue samples, the cells were stained on their surfaces, followed by detection of the relevant lymphocytes using a flow cytometer.

The colonic contents were dissected, and approximately 200 mg of fresh fecal samples were collected into sterile EP tubes and immediately snap-frozen in liquid nitrogen, then transferred to a -80°C freezer for storage. Collected tissues underwent histopathological staining, including hematoxylin-eosin (HE) and oil red O staining for the liver. In addition, fecal samples were subjected to gut microbiota analysis, and liver tissues were analyzed using untargeted metabolomics.

#### Methods for quantitative profiling of fecal bile acids

An appropriate amount of fecal samples from humans or rats was precisely weighed into a 2 mL centrifuge tube, followed by the addition of stainless-steel beads and 1 mL of a pre-cooled extraction solvent mixture. The mixture was vigorously vortexed for 30 s to ensure comprehensive homogenization. The sample was subsequently homogenized at 25 Hz for 10 min, followed by ultrasonic-assisted extraction in an ice-water bath for 10 min, and then rigorously vortexed for an additional 5 min. After extraction, the mixture was centrifuged at 12,000 rpm for 10 min at 4°C. Finally, 500 μL of the resulting supernatant was filtered through a 0.22 μm membrane to remove particulate matter (tiny solid or liquid particles). Bile acids were quantitatively profiled by ultra-high-performance liquid chromatography-tandem triple-quadrupole mass spectrometry (UHPLC-MS/MS), a targeted metabolomic platform with validated sensitivity and precision. The analytical platform comprises Vanquish™ UHPLC system (Thermo Fisher Scientific Inc., Waltham, MA, USA) and Orbitrap Exploris™ 120 high-resolution mass spectrometer (Thermo Fisher Scientific Inc., Bremen, Germany). Chromatographic separation of target compounds was performed on a Waters ACQUITY I-Class ultra-high-performance liquid chromatography system equipped with an ACQUITY UPLC BEH C18 column. The mobile phase consisted of solvent A (0.1% formic acid in water with 5 mM ammonium acetate) and solvent B (acetonitrile). The analytical conditions were precisely controlled, with the column temperature maintained at 45°C, the autosampler temperature stabilized at 4°C, and a consistent injection volume of 2 μL. Mass spectrometry analysis was performed using an Orbitrap Exploris 120 high-resolution mass spectrometer, specifically configured in parallel reaction monitoring (PRM) mode for enhanced metabolomic profiling. The ion source parameters were as follows: Spray voltage = +3500/-3200 V, Sheath gas (N2) flow rate = 40, Aux gas (N2) flow rate = 15, Sweep gas (N2) flow rate = 0, Aux gas (N2) temperature = 350 °C, Capillary temperature = 320 °C.

#### Methods for microbial diversity 16S rRNA gene amplicon sequencing

Microbial community genomic DNA was extracted from fecal samples. The integrity of the extracted DNA was assessed by 1% agarose gel electrophoresis, and DNA concentration and purity were determined using a NanoDrop 2000 spectrophotometer (Thermo Scientific, USA). The V3-V4 hypervariable regions of the 16S rRNA gene were amplified using the forward primer 338F (5′-ACTCCTACGGGAGGCAGCAG-3′) and the reverse primer 806R (5′-GGACTACHVGGGTWTCTAAT-3′), both tagged with unique barcodes. Polymerase Chain Reaction (PCR) was performed using the following conditions: initial denaturation at 95 °C for 3 min; 27 cycles of denaturation at 95 °C for 30 s, annealing at 55 °C for 30 s, and extension at 72 °C for 30 s; followed by a final extension at 72 °C for 10 min; and storage at 4 °C. The PCR reaction mixture contained: 4 μL of 5× TransStart FastPfu buffer, 2 μL of 2.5 mM dNTPs, 0.8 μL of 5 μM forward primer, 0.8 μL of 5 μM reverse primer, 0.4 μL of TransStart FastPfu DNA polymerase, and 10 ng of template DNA, with ddH_2_O added to a final volume of 20 μL. PCR products from the same sample were pooled and purified using 2% agarose gel electrophoresis. The band size was verified, and the purified products were quantified using a Synergy HTX microplate reader (BioTek, USA). Library preparation was conducted using the NEXTFLEX Rapid DNA-Seq Kit (Bioo Scientific, Austin, Texas, USA), which involved adaptor ligation, bead-based size selection to remove self-ligated fragments, PCR enrichment, and magnetic bead recovery. The final libraries were sequenced on the Illumina NextSeq 2000 platform using paired-end 300 bp reads. Raw paired-end reads were quality-filtered using fastp (v0.19.6, https://github.com/OpenGene/fastp) and merged with FLASH (v1.2.11, http://www.cbcb.umd.edu/software/flash). Denoising was performed on quality-filtered sequences using the DADA2 plugin in Qiime2 under default parameters, resulting in amplicon sequence variants (ASVs). Taxonomic classification of ASVs was conducted using a Naive Bayes (or Vsearch, or Blast) classifier in Qiime2 based on the Silva 16S rRNA gene database (v138).

### Quantification and statistical analysis

#### Analysis of differential fecal bile acids

Bile acid profiles were determined in fecal samples from humans (*n* = 49) and rats (*n* = 11). Differential bile acids were identified through the Mann-Whitney U test, with statistical significance defined as a *P*-value less than 0.05. Normalized levels of various bile acids in human fecal samples are displayed by heatmaps. Bile acids in different categories of rat feces are expressed as mean ± Standard Error of the Mean (SEM).

#### Analysis of microbial diversity 16S rRNA gene amplicon sequencing

Microbial diversity 16S rRNA gene amplicon sequencing were performed to comprehensively characterize the microbial diversity in fecal samples obtained from both humans (*n* = 73) and rats (*n* = 29). Functional prediction of microbial communities was carried out using PICRUSt2 (v2.2.0). Between-group comparisons were made using the Wilcoxon rank-sum test, and the Bray-Curtis distance algorithm was applied for principal coordinate analysis (PCoA) to assess the similarity of microbial community structures among samples. Additionally, the PERMANOVA nonparametric test was utilized to determine the significance of differences in microbial community structures between sample groups. The relative abundance of differential gut microbiota in human fecal samples are expressed as median (interquartile range). The relative abundance of differential gut microbiota in rat fecal samples are expressed as mean difference with 95 % confidence interval (95 % CI). To identify significant differences in bacterial abundance between groups, Linear discriminant analysis Effect Size (LEfSe) method was employed. This approach systematically identified differentially abundant bacterial taxa from phylum to genus level, with statistical significance defined by LDA score >2 and *P*<0.05.

#### Statistical analysis

Normally distributed data is expressed as mean with standard deviation (SD) and analyzed via t-tests or one-way analysis of variance (ANOVA). For skewed data, results were presented as median (interquartile range, P_25_-P_75_), with group comparisons performed using nonparametric statistical methods. Categorical variables were represented as frequencies (percentages), and comparisons between groups were analyzed using the chi-square test. Correlation analyses were conducted using Pearson’s correlation for normally distributed data and Spearman’s rank correlation for non-normally distributed or skewed data, with normality assessed using the Shapiro-Wilk test and visual inspection of Q-Q plots. Protein band intensities were quantified using ImageJ software after converting the Western blot images to 8-bit grayscale, subtracting background and measuring the integrated density (IntDen) within identical rectangular regions of interest. The relative expression level of each target protein was normalized to its corresponding loading control. GraphPad Prism 8.0.2 software was also employed for partial statistical plotting. A two-sided *P*-value of less than 0.05 was considered statistically significant. All statistical analyses were performed using R software version 4.4.2 (R Foundation for Statistical Computing).

### Additional resources

Clinical trial registry numbers and Clinical trial registry URL: ChiCTR2500096000 (https://www.chictr.org.cn/showproj.html?proj=239950).

## Supplemental information

Document S1. Figure S1 and S2

Table S1. Demographic characteristics of participants in the case-control study, related to Figure 2

Table S2. Comparison of different classes of bile acids in human fecal samples, related to Figure 3

Table S3. Feed ingredient content for animal experiment, related to Figure 4
