# Supplementary material for: Integrative analysis of the gut microbiota, bile acid pathways, and immune dysregulation in dyslipidemia models
Source: iScience. 2025 Nov 10;28(12):114001. doi: 10.1016/j.isci.2025.114001 (PMC12686710; doi:10.1016/j.isci.2025.114001)

iScience, Volume *28*

Supplemental information

Integrative analysis of the gut microbiota, bile acid pathways, and immune dysregulation in dyslipidemia models

Jiayue Xia, Yinqi Shao, Boxuan Li, Tianyu Wu, Zhi He, Zhiyuan Feng, Zhenzheng Zhang, Shiyu Yin, Yuanyuan Wang, Junhui Yu, Jiongnan Wang, and Guiju Sun

Supplementary Figure S1: Original protein bands

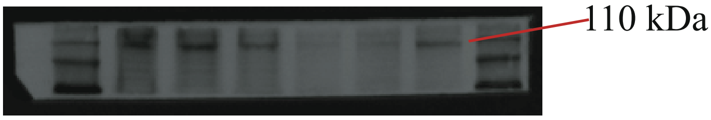

FGFR4 protein bands

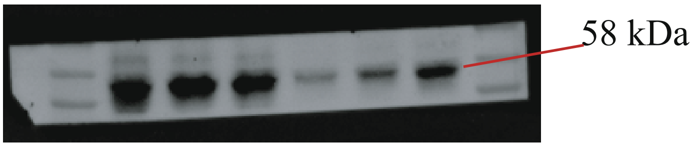

CYP7A1 protein bands

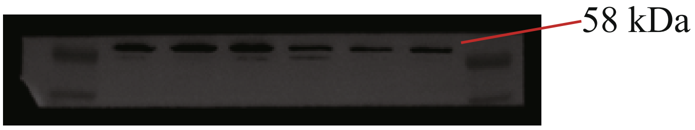

CYP8B1 protein bands

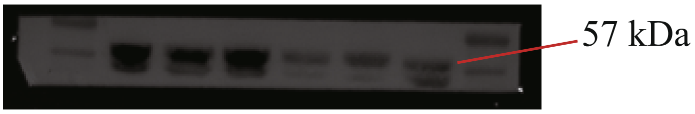

FXR protein bands

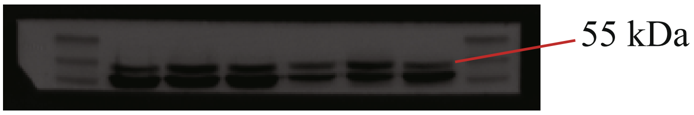

CYP27A1 protein bands

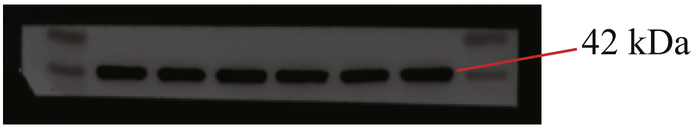

β-Actin protein band

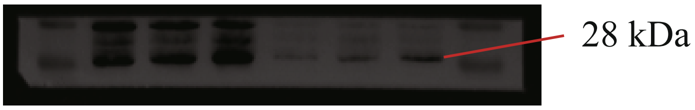

SHP protein band

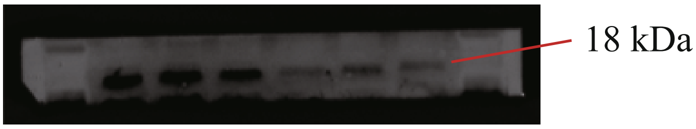

FGF15 protein bands

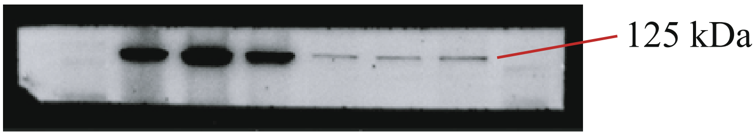

SREBP-1 protein band

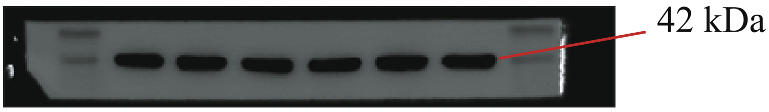

β-Actin protein band for SREBP-1

Supplementary Figure S2: Heatmap of Spearman's correlation analysis between fecal bile acids and gut microbiota in human participants

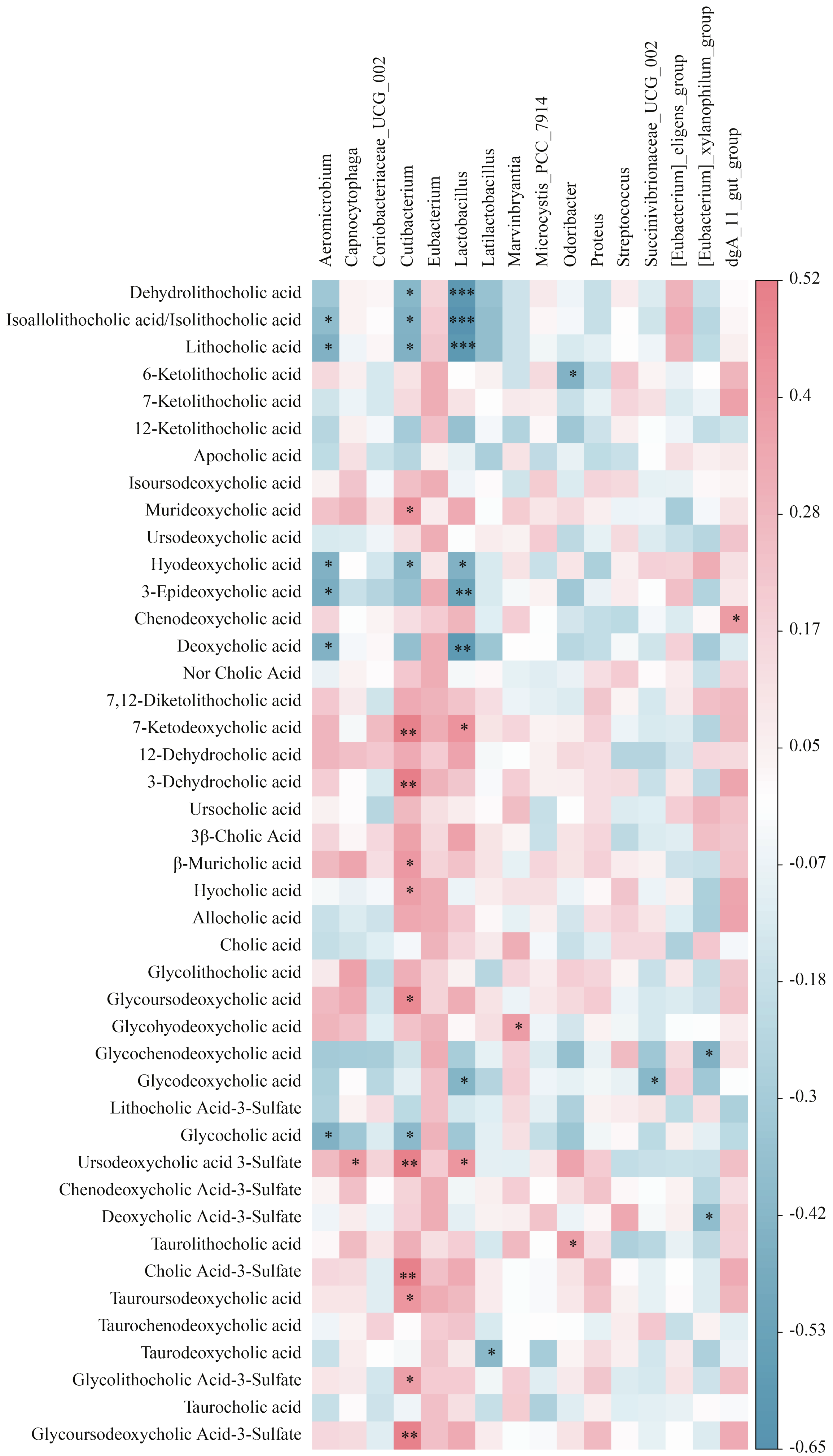

Supplement: Document S1. Figure S1 and S2 [file mmc1.pdf]
